# Efficient synthesis and antimicrobial evaluation of some Mannich bases from 2-arylidine-1-thia-4-azaspiro[4.5]decan-3-ones

**DOI:** 10.1186/s13065-015-0101-8

**Published:** 2015-05-10

**Authors:** Essam M Hussein, Ghada S Masaret, Khalid S Khairou

**Affiliations:** Department of Chemistry, Faculty of Applied Sciences, Umm Al-Qura University, Makkah, Saudi Arabia; Department of Chemistry, Faculty of Science, Assiut University, Assiut, 71516 Egypt

**Keywords:** Spiro, 1-thia-4-azaspiro[4.5]decan-3-one, Sodium dodecylbenzene sulfonate, Antimicrobial activity, Mannich bases

## Abstract

**Background:**

Thiazolidinone, has been employed in the preparation of different important drugs required for treatment of inflammations, bacterial infections, and hypertension. Mannich bases have been shown to exhibit diverse biological activities, such as antibacterial, and antifungal activities. Spiroheterocycles including thiazolidine moiety have antimicrobial activity.

**Results:**

In this study, a novel, rapid, and efficient protocol is developed for the synthesis of various 2-arylidine-1-thia-4-azaspiro[4.5]decan-3-ones using sodium dodecylbenzene sulfonate (DBSNa) as an inexpensive and readily available reagent in acetic acid at room temperature. High yields, easy work-up, and short reaction times are advantages of this procedure. The synthesized arylidines were undergone Mannich reaction with formaldehyde and secondary amines in absolute ethanol at room temperature to afford the corresponding N-Mannich bases. All prepared Mannich bases were evaluated for their antimicrobial activity.

**Conclusions:**

Good activity was noted for Mannich bases from 2-arylidine-1-thia-4-azaspiro[4.5]decan-3-ones, with some members recorded higher antimicrobial activity.

**Graphical abstract:**
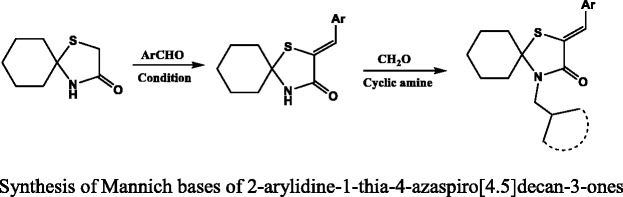
Synthesis of Mannich bases of 2-arylidine-1-thia-4-azaspiro[4.5]decan-3-ones.

## Background

There are many bioactive molecules which possess various heteroatoms such as nitrogen, sulfur and oxygen, always taken the attention of chemists over the years mainly because of their biological significance. Thiazolidinones are thiazolidine derivatives which have a sulfur atom at position 1, a nitrogen atom at position 3 and a carbonyl group at position 2, 4, or 5 [1], is considered as an important biologically active scaffold that possesses almost all types of biological activities [2]. This heterocyclic system has been employed in the preparation of different important drugs required for treatment of inflammations [3], bacterial infections [4], and hypertension [5]. Some of the thiazole analogues are used as fungicides, inhibiting in vivo the growth of xanthomonas and as ingredients of herbicides, antischistosomicidal, and anthelmintic drugs [6]. Mannich bases are reported to show a diversity of biological activities, such as antibacterial [7,8], antifungal [9,10] activities. Spiro derivatives have antibacterial, anticancer, and anticonvulsants activities. Spiro heterocycles were used as nitric oxide synthesis inhibitors [11] and potential topical agents for vaginal infection [12]. Spiro heterocyclic compounds including thiazolidine moiety have antimicrobial activity [13].

In this paper and as a consequence of our previous work on the synthesis of *N*-heterocyclic compounds [14–18], and bioactive heterocyclic agents [19–21], we reported herein an efficient protocol to the synthesis of *N*-Mannich bases (**6a-r**) from 2-arylidine-1-thia-4-azaspiro[4.5]decan-3-ones (**5a-f**). The anti-microbial activity of the prepared compounds (**6a-r**) was screened.

## Results and discussion

### Chemistry

**Scheme 1:**
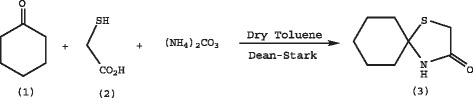
Synthesis of 1-thia-4-azaspiro[4.5]decan-3-one (**3**).

First of all, 1-thia-4-azaspiro[4.5]decan-3-one (**3**) was prepared via the three component cyclocondensation reaction of cyclohexanone, thioglycolic acid, and ammonium carbonate according to the previously reported procedure [22] as shown in Scheme 1.

#### Synthesis of 2-arylidine-1-thia-4-azaspiro[4.5]decan-3-ones (5a-f)

**Scheme 2:**
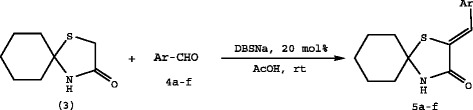
Synthesis of 2-arylidine-1-thia-4-azaspiro[4.5]decan-3-ones (**5a-f**).

In the first part of our research, we investigated a novel, rapid and efficient protocol that was developed for the synthesis of some 2-arylidine-1-thia-4-azaspiro[4.5]decan-3-ones (**5a**–**f**) by the condensation of 1-thia-4-azaspiro[4.5]decan-3-one (**3**) with aromatic aldehydes (**4a**–**f**) using sodium dodecylbenzene sulfonate (DBSNa) (20 mol %) in acetic acid at room temperature as shown in Scheme 2 and Table 1.

**Table 1:** **Synthesis of the 2-arylidine-1-thia-4-azaspiro[4.5]decan-3-ones (5a–f) using DBSNa (15 mol%)**

**Entry**	**Product** ^**a**^	**Ar**	**Time (min)**	**Yield** ^**b**^ **(%)**
1	**5a**	Ph	60	94
2	**5b**	4-ClC_6_H_4_	50	96
3	**5c**	4-BrC_6_H_4_	60	93
4	**5d**	4-O_2_NC_6_H_4_	45	87
5	**5e**	4-MeOC_6_H_4_	40	98
6	**5f**	4-pyridyl	70	90

^a^ Reaction conditions: 1-thia-4-azaspiro[4.5]decan-3-one (**3**) (10 mmol), aromatic aldehydes (**4a-f**) (10 mmol), and DBSNa (20 mol%) in 10 mL acetic acid at room temperature.

^b^ Isolated yields.

**Scheme 3:**
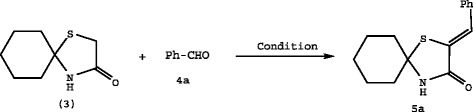
Synthesis of 2-benzylidine-1-thia-4-azaspiro[4.5]decan-3-ones (**5a**).

To find out the suitable conditions for the synthesis of 2-arylidine-1-thia-4-azaspiro[4.5]decan-3-ones, a series of experiments were performed with the standard reaction of 1-thia-4-azaspiro[4.5]decan-3-one (**3**) and benzaldehyde (**4a**) as a model reaction (Scheme 3, Table 2).

**Table 2:** The effect of reaction condition on the synthesis of (5a) under various conditions^**a**^

**Entry**	**Solvent** ^**b**^	**Catalyst** ^**c**^	**Time (min)**	**Yield** ^**d**^ **(%)**
1	EtOH	Piperidine	180	37
2	EtOH	AcOH	180	43
3	EtOH	*p*-TSA	180	40
4	AcOH	AcONa	150	46
5	AcOH	H_2_SO_4_	120	61
6	EtOH	DBSNa	120	66
7	MeOH	DBSNa	120	64
8	AcOH	DBSNa	60	94
9	H_2_O	DBSNa	180	trace

^a^ The reaction was carried out with 1-thia-4-azaspiro[4.5]decan-3-one (**3**) (10 mmol), benzaldehydes (**4a**) (10 mmol) at room temperature.

^b^ 10 mL solvent.

^c^ 20 mol%.

^d^ Isolated yields.

##### Effect of the reaction conditions

In our initial study, we tried to optimize the model procedure mentioned above by detecting the efficiency of different reaction conditions, such as piperidine/EtOH, *p*-TSA/EtOH, AcOH/EtOH, AcONa/AcOH, H_2_SO_4_/AcOH, DBSNa/MeOH, DBSNa/EtOH, DBSNa/H_2_O, DBSNa/AcOH (Table 2).

In each case, the reactants (10 mmol) were allowed together in 10 mL solvent at room temperature. In the case of piperidine/EtOH, *p*-TSA/EtOH, AcOH/EtOH, and AcONa/AcOH, the reaction proceeded with comparatively longer reaction time and poor reaction yield (Table 2, entries 1–4). Acetic acid acidified with a drop of H_2_SO_4_ can push the reaction towards the formation of product in yields of 61% (Table 2, entry 5).

In the presence of sodium dodecylbenzene sulfonate (DBSNa), the reaction was possible and the product (**5a**) was obtained in good yields.

Sodium dodecylbenzene sulfonate was used in different reaction media such as ethanol, methanol, water, and acetic acid (Table 2, entries 6–9). The best results were obtained when DBSNa was used as catalyst in acetic acid as reaction medium, which provided a yield of 94% (Table 2, entry 8).

Unfortunately, when the reaction was performed in water, the yield of the desired product was obtained in a trace amount (Table 2, entry 9).

##### Evaluation of catalytic activity of DBSNa

To determine the appropriate concentration of the catalyst used, we investigated the model reaction at different concentrations of DBSNa (5, 10, 15, 20, and 25 mol %). It was found that when the amount of DBSNa was increased from 5 to 20 mol%, the yield increased from 68 to 94%, respectively. However, there was no significant change in reaction yield when the amount of catalyst was increased further, to 25 mol%. Thus, 20 mol% DBSNa in acetic acid is sufficient to push this reaction forward (Table 3).

**Table 3:** Evaluation of catalytic activity of DBSNa in the synthesis of (5a)^**a**^

**Entry**	**Amount of DBSNa (mol %)**	**Time (min)**	**Yield** ^**b**^ **(%)**
1	5	90	68
2	10	70	81
3	15	60	87
4	20	60	94
5	25	60	94

^a^ The reaction was carried out with 1-thia-4-azaspiro[4.5]decan-3-one (**3**) (10 mmol), benzaldehydes (**4a**) (10 mmol) and DBSNa in 10 mL acetic acid at room temperature.

^b^ Isolated yields.

#### Synthesis of Mannich bases of 2-arylidine-1-thia-4-azaspiro[4.5]decan-3-ones (6a-r)

**Scheme 4:**
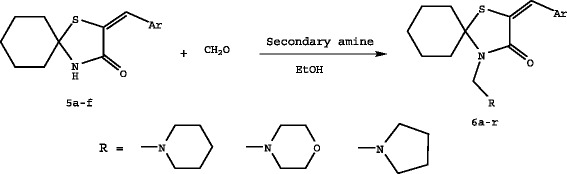
Synthesis of Mannich bases (**6a-r**).

The second part of the research includes the preparation of a series of Mannich bases (6a-r) in good yield (71-91%) by the reaction of 2-arylidine-1-thia-4-azaspiro[4.5]decan-3-ones (**5a**–**f**) with formaldehyde and secondary amines (piperidine, morpholine, and pyrrolidine) in absolute ethanol at room temperature for 1.5-4 h (Scheme 4 and Table 4).

**Table 4:** Synthesis of the Mannich bases (6a-r)^a^

**Mannich product**	**Ar**	**R**	**Time (h)**	**Yield** ^**b**^ **(%)**
**6a**	Ph	piperidin-1-yl	2.0	80
**6b**	Ph	morphilin-1-yl	3.0	76
**6c**	Ph	pyrrolidin-1-yl	2.5	79
**6d**	4-ClC_6_H_4_	piperidin-1-yl	1.5	91
**6e**	4-ClC_6_H_4_	morphilin-1-yl	2.5	88
**6f**	4-ClC_6_H_4_	pyrrolidin-1-yl	2.0	80
**6g**	4-BrC_6_H_4_	piperidin-1-yl	2.0	81
**6h**	4-BrC_6_H_4_	morphilin-1-yl	2.0	80
**6i**	4-BrC_6_H_4_	pyrrolidin-1-yl	3.0	80
**6j**	4-O_2_NC_6_H_4_	piperidin-1-yl	3.0	76
**6k**	4-O_2_NC_6_H_4_	morphilin-1-yl	4.0	71
**6l**	4-O_2_NC_6_H_4_	pyrrolidin-1-yl	3.5	73
**6m**	4-MeOC_6_H_4_	piperidin-1-yl	1.5	89
**6n**	4-MeOC_6_H_4_	morphilin-1-yl	2.5	81
**6o**	4-MeOC_6_H_4_	pyrrolidin-1-yl	2.0	83
**6p**	4-pyridyl	piperidin-1-yl	2.0	89
**6q**	4-pyridyl	morphilin-1-yl	3.0	79
**6r**	4-pyridyl	pyrrolidin-1-yl	2.0	82

^a^ Reaction conditions: 2-arylidine-1-thia-4-azaspiro[4.5]decan-3-ones (**5a-f**) (10 mmol), formaldehyde (15 mmol), and secondary amine (15 mmol) in 10 mL absolute ethanol at room temperature.

^b^ Isolated yields.

The structures of the isolated new products (**6a-r**) were deduced by analyzing their physical and spectroscopic data, such as the data obtained using IR, ^1^H NMR, and ^13^C NMR spectroscopy.

Taking (**6b**) as an example, the IR spectrum showed the lack of the absorption band corresponding to NH group. The ^1^H NMR spectrum showed the presence of a singlet signal at δ = 4.15 ppm for the methylene protons and two triplet signals at 2.47, 3.59, ppm for morpholine ring protons.

### Antimicrobial activity

#### In vitro antibacterial activity

The synthesized compounds (**6a-r**) were screened in vitro for their antibacterial at 50 mg/mL concentration against *Staphylococcus aureus* as Gram positive bacteria, *Escherichia Coli*, *Pseudomonas aeroginosa* as Gram negative bacteria using Ciprofloxacin as standard antibacterial reference. Most of the tested compounds showed excellent antibacterial activities with respect to the reference drug.

The results obtained in Table 5 indicated that the type of substituents (*Ar*) and (*R*) are the controlling factors in developing the total antibacterial properties of the tested Mannich bases (**6a-r**).

**Table 5:** Bactericidal activity of Mannich bases (6a-r) using Ciprofloxacin as standard antibacterial reference^a^

**Compound**	***Staphylococcus aureus***	***Escherichia Coli***	***Pseudomonas aeroginosa***
**6a**	--	+	--
**6b**	++	+	++
**6c**	--	--	--
**6d**	++	+	+
**6e**	+++	+++	+++
**6f**	--	+	--
**6g**	+	++	--
**6h**	++	++	++++
**6i**	--	--	+
**6j**	++	--	--
**6k**	+	+++	++
**6l**	--	--	--
**6m**	+++	+++	++
**6n**	++++	++++	+++
**6o**	+	+	--
**6p**	++++	+	++
**6q**	++++	+++	+++
**6r**	-	++	+
**Ciprofloxacin**	++++	++++	++++

^a^ The activities are based on the diameter of zones of inhibition in mm. 50 μL of stock solution was applied in each hole of each paper disk. +: <15 mm; ++: 15–24 mm; +++: 25–34 mm; ++++: 35–44 mm.

Data in Table 5 revealed that compounds **6e**, **6 h**, **6 k**, **6 m**, **6n**, **6p** and **6q**, have superior significant antibacterial potency. Compounds **6n** and **6q** [*Ar* = 4-MeOC_6_H_4_ and 4-pyridyl; *R* = morpholin-1-yl] have excellent activities against Gram positive bacteria (*Staphylococcus aureus*) and Gram-negative bacteria (*Escherichia Coli* and *Pseudomonas aeroginosa*). Compound **6p** [*Ar* = 4-pyridyl; *R* = piperidin-1-yl] has excellent potency against *Staphylococcus aureus*, moderate activity against *Pseudomonas aeroginosa*, and poor activity against *Escherichia Coli*. Structure-Activity relationships (SAR) based on the obtained results indicated that the best observed antibacterial activity is that which *Ar* is phenyl ring attached with electron donating function (MeO) as exhibit in compound **6n**. However, substituting the phenyl ring with electron withdrawing function (Cl, NO_2_) the antibacterial behavior is decreased. When *Ar* is unsubstituted phenyl ring the antibacterial activity is not significant. It has also, been noticed that when *Ar* is pyridine ring exhibited in (**6p**) and (**6q**) increase in the observed antibacterial properties was noticed compared with the case of using phenyl ring.

#### In vitro antifungal activity

With respect to antifungal activity, the synthesized compounds were screened against three fungal strains; *Aspergillus niger*, *Candida albicans, Fusarium oxysporium* using Nystatin as standard antifungal reference (at 50 mg/mL concentration). Most of the tested compounds showed excellent antibacterial activities with respect to the reference drug.

As antibacterial activity, the obtained results indicated that the type of substituents (*Ar*) and (*R*) are the controlling factors in developing the antifungal properties of the tested compounds (**6a-r**). Results of antifungal activities were shown in Table 6. Data in Table 6 showed that compounds **6d**, **6 l**, and **6p** have remarkable antifungal potency. Compounds **6 l** [*Ar* = 4-O_2_NC_6_H_4_; *R* = piperidin-1-yl] exhibit excellent activities against *Candida albicans* and *Fusarium oxysporium* as well as good potency against *Aspergillus niger*. Compound **6p** [*Ar* = 4-pyridyl; *R* = piperidin-1-yl] has excellent activity against *Aspergillus niger* as well as good potency against *Candida albicans* and *Fusarium oxysporium*. Compound **6d** [*Ar* = 4-ClC_6_H_4_; *R* = piperidin-1-yl] exhibit good potency against *Aspergillus niger*, *Candida albicans* and *Fusarium oxysporium*. Structure-Activity relationships (SAR) based on the obtained results indicated that the best observed antifungal activity is that which *Ar* is phenyl ring attached with electron withdrawing function (NO_2_, Cl) as exhibit in compound **6l** and **6d**, respectively. However, substituting the phenyl ring with electron donating function (MeO) the antifungal behavior is decreased. When *Ar* is unsubstituted phenyl ring the antifungal activity is not remarkable. It has also, been noticed that when *Ar* is pyridine ring exhibited in (**6p**) increase in the observed potent antifungal properties was noticed compared with the case of using phenyl ring.

**Table 6:** Fungicidal activity of Mannich bases (6a-r) using Nystatin as standard antifungal reference^a^

**Compound**	***Aspergillus niger***	***Candida albicans***	***Fusarium oxysporium***
**6a**	+	+	+
**6b**	+	--	+
**6c**	--	--	--
**6d**	+++	+++	+++
**6e**	++	+++	+
**6f**	+	++	--
**6g**	++	++	++
**6h**	+	+	--
**6i**	--	++	--
**6j**	++	-	+
**6k**	++	+++	++
**6l**	+++	++++	++++
**6m**	++	--	--
**6n**	+	+	++
**6o**	+++	++	--
**6p**	++++	+++	+++
**6q**	+++	++	+++
**6r**	+	+	+
**Nystatin**	++++	++++	++++

^a^ The activities are based on the diameter of zones of inhibition in mm. 50 μL of stock solution was applied in each hole of each paper disk. +: <15 mm; ++: 15–24 mm; +++: 25–34 mm; ++++: 35–44 mm.

## Conclusions

The authors have developed a novel, rapid and efficient protocol for the synthesis of various 2-arylidine-1-thia-4-azaspiro[4.5]decan-3-ones using sodium dodecylbenzene sulfonate (DBSNa) in acetic acid at room temperature. The results clearly demonstrate that the using of the sodium dodecylbenzene sulfonate as an inexpensive and readily available reagent markedly enhances the efficiency of the chemical processes of interest. Mannich bases from the synthesized 2-arylidine-1-thia-4-azaspiro[4.5]decan-3-ones were achieved and evaluated as antimicrobial agents and showed remarkable activities.

### Experimental

#### Chemistry

##### General methods

The time required for completion of each reaction was monitored by TLC. All melting points are uncorrected and were measured on a Gallenkamp apparatus. The IR spectra were recorded on a Shimadzu 470 IR spectrometer (KBr) νmax, cm^−1^. The ^1^H and ^13^C NMR spectra were measured on a Varian EM-200 (1H: 400 MHz, ^13^C: 100 MHz) spectrometer with TMS as internal standard. Mass spectra were determined on a JEOL JMS-600 spectrometer. Elemental analyses (C, H, N, and S) were performed on an elemental analysis system GmbH VarioEL V_2.3_.

### General procedure for synthesis of 2-Arylidine-1-thia-4-azaspiro[4.5]decan-3-ones (5a-f)

To a solution of (**3**) (1.71 g, 10 mmol) in acetic acid (10 mL), corresponding aromatic aldehyde (10 mmol) was added. Then DBSNa (20 mol%) was added and the reaction mixture was stirred at room temperature for the desired time as monitored by TLC (Table 1). After completion of the reaction, the solid product was filtered and washed with cold water, dried, and recrystallized from ethanol (95%).

### 2-Benzylidene-1-thia-4-azaspiro[4.5]decan-3-one (5a)

Pale yellow crystals; mp 200–202°C; IR: 3200 (NH), 3020 (CH arom.), 1700 (C = O), 695 (C-S); ^1^H NMR (DMSO-d_6_): 1.10–1.95 (m, 10H, 5 × CH_2_), 7.50 (m, 5H, Ph-H), 7.85 (s, 1H, CH), 8.97 (s, 1H, NH, D_2_O-exchangeable) ppm; ^13^C NMR: 21.7 (2 CH_2_), 24.8 (CH_2_), 25.6 (2CH_2_), 47.9 (spiro C), 122.3 (CH), 124.8 (2CH), 126.1 (2CH), 129.4 (C), 131.1 (C), 136.7 (C), 170.2 (C = O) ppm; EI-MS, *m*/*z* (%): 260.10 (8) [M + 1], 259.01 (54) [*M*+], 216.05 (100.0). Calc. for C_15_H_17_NOS: C, 69.46; H, 6.61; N, 5.40; S, 12.36. Found: C, 69.28; H, 6.47; N, 5.24; S, 12.16.

### 2-(4-Chlorobenzylidene)-1-thia-4-azaspiro[4.5]decan-3-one (5b)

Pale yellow crystals; mp 210–212°C; IR: 3190 (NH), 3085 (CH arom.), 1695 (C = O), 695 (C-S); ^1^H NMR (DMSO-d_6_): 1.15–2.10 (m, 10H, 5 × CH_2_), 7.35 (d, 2H, 2CH, *J* = 7.4), 8.00 (d, 2H, 2CH, *J* = 7.4), 8.90 (s, 2H, CH + NH) ppm; EI-MS, *m*/*z* (%): 293.54 (44) [*M*+], 258.15 (30), 250.02 (100.0). Calc. for C_15_H_16_ClNOS: C, 61.32; H, 5.49; Cl, 12.07; N, 4.77; S, 10.91. Found: C, 61.12; H, 5.30; Cl, 11.88; N, 4.59; S, 10.78.

### 2-(4-Bromobenzylidene)-1-thia-4-azaspiro[4.5]decan-3-one (5c))

Pale yellow crystals; mp 221–222°C; IR: 3200 (NH), 3090 (CH arom.), 1695 (C = O), 695 (C-S); ^1^H NMR (DMSO-d_6_): 1.00–2.10 (m, 10H, 5 × CH_2_), 7.40 (d, 2H, 2CH, *J* = 7.2), 8.10 (d, 2H, 2CH, *J* = 7.2), 8.60 (s, 1H, CH), 10.10 (s, 1H, NH) ppm; EI-MS, *m*/*z* (%): 339.01 (20) [*M*+], 296.15 (100.0), 79 (15). Calc. for C_15_H_16_BrNOS: C, 53.26; H, 4.77; Br, 23.62; N, 4.14; S, 9.48. Found: C, 53.00; H, 4.80; Br, 23.47; N, 3.81; S, 9.19.

### 2-(4-Nitrobenzylidene)-1-thia-4-azaspiro[4.5]decan-3-one (5d))

Yellow crystals; mp 249–250°C; IR: 3220 (NH), 3090 (CH arom.), 1690 (C = O), 690 (C-S); ^1^H NMR (DMSO-d_6_): 1.20–2.10 (m, 10H, 5 × CH_2_), 8.10 (d, 2H, 2CH, *J* = 7.4), 8.50 (d, 2H, 2CH, *J* = 7.4), 8.70 (s, 1H, CH), 10.20 (s, 1H, NH) ppm; EI-MS, *m*/*z* (%): 304.61 (29) [*M*+], 261.25 (100.0). Calc. for C_15_H_16_N_2_O_3_S: C, 59.19; H, 5.30; N, 9.20; S, 10.54. Found: C, 58.89; H, 5.43; N, 9.00; S, 10.30.

### 2-(4-Methylbenzylidene)-1-thia-4-azaspiro[4.5]decan-3-one (5e))

Yellow crystals; mp 230–231°C; IR: 3200 (NH), 3090 (CH arom.), 1695 (C = O), 690 (C-S); ^1^H NMR (DMSO-d_6_): 1.10–2.05 (m, 10H, 5 × CH_2_), 3.85 (s, 3H, CH_3_), 7.05 (d, 2H, 2CH, *J* = 7.3), 7.60 (d, 2H, 2CH, *J* = 7.3), 7.85 (s, 1H, CH), 8.90 (s, 1H, NH) ppm; ^13^C NMR: 22.2 (2 CH_2_), 24.7 (CH_2_), 26.1 (2CH_2_), 42.1 (CH_3_), 47.3 (spiro C),121.9 (CH), 125.3 (2CH), 126.7 (2CH), 129.2 (C), 131.5 (C), 136.9 (C), 171.3 (C = O) ppm; EI-MS, *m*/*z* (%): 290.54 (12) [*M*^+^ + 1], 289.02 (40) [*M*^+^], 274.01 (17), 246.12 (100.0). Calc. for C_16_H_19_NO_2_S: C, 66.41; H, 6.62; N, 4.84; S, 11.08. Found: C, 66.09; H, 6.45; N, 4.68; S, 10.85.

### 2-(Pyridin-4-ylmethylene)-1-thia-4-azaspiro[4.5]decan-3-one (5f))

Pale yellow crystals; mp 225–227°C; IR: 3190 (NH), 3090 (CH arom.), 1695 (C = O), 695 (C-S); ^1^H NMR (DMSO-d_6_): 1.05–2.10 (m, 10H, 5CH_2_), 7.00 (d, 2H, 2CH, *J* = 6.8), 7.70 (d, 2H, 2CH, *J* = 6.8), 7.80 (s, 1H, CH), 8.10 (s, 1H, NH) ppm; EI-MS, *m*/*z* (%):260.15 (25) [*M*+], 217.15 (100.0). Calc. for C_14_H_16_N_2_OS: C, 64.58; H, 6.19; N, 10.76; S, 12.32. Found: C, 64.36; H, 6.00; N, 10.79; S, 12.20.

### General procedure for synthesis of Mannich Bases (6a-r))

To a solution of 2-benzylidene-1-thia-4-azaspiro[4.5]decan-3-one **(5a)** (0.259 g, 1 mmol) in 5 mL of absolute ethanol was added a mixture of sec. amine (1.5 mmol) and aqueous formaldehyde 35% (0.2 mL, 1.5 mmol) also dissolved in 5 mL absolute ethanol. The reaction mixture was stirred for the desired time as monitored by TLC (Table 4), refrigerated for 24 h to form crystals. The crystalline product was separated by filtration, vacuum dried and recrystallized from ethanol.

### 2-Benzylidene-4-(piperidin-1-ylmethyl)-1-thia-4-azaspiro[4.5]decan-3-one (6a))

Pale yellow crystals; mp 119–120°C; IR: 3030 (CH arom.), 2950 (CH aliph.), 1700 (C = O), 695 (C-S); ^1^H NMR (CDCl_3_): 1.38–1.51 (m, 12H, 6CH_2_), 2.10-2.37 (m, 4H, 2CH_2_), 2.43-2.48 (m, 4H, 2CH_2_), 4.08 (s, 2H, CH_2_), 7.32-7.60 (m, 5H, Ph-H), 7.75 (s, 1H, CH) ppm; EI-MS, *m*/*z* (%): 357.05 (9) [M^+^ + 1], 356.01 (46) [*M*^*+*^], 258.12 (25), 215.65 (100.0). Calc. for C_21_H_28_N_2_OS: C, 70.75; H, 7.92; N, 7.86; S, 8.99. Found: C, 70.52; H, 7.80; N, 7.66; S, 8.78.

### 2-Benzylidene-4-(morpholinomethyl)-1-thia-4-azaspiro[4.5]decan-3-one (6b))

Pale yellow crystals; mp 135–137°C; IR: 3020 (CH arom.), 2955 (CH aliph.), 1700 (C = O), 695 (C-S); ^1^H NMR (DMSO-d_6_): 1.42–1.51 (m, 6H, 3CH_2_), 2.15-2.40 (m, 4H, 2CH_2_), 2.47 (t, 4H, 2CH_2_, *J* = 6.9), 3.59 (t, 4H, 2CH_2_, *J* = 6.9), 4.15 (s, 2H, CH_2_), 7.33-7.61 (m, 5H, Ph-H), 7.71 (s, 1H, CH) ppm; EI-MS, *m*/*z* (%): 359.01 (5) [M^+^ + 1], 358.00 (32) [*M*^*+*^], 258.09 (17), 216.05 (100.0). Calc. for C_20_H_26_N_2_O_2_S: C, 67.01; H, 7.31; N, 7.81; S, 8.94. Found: C, 66.80; H, 7.05; N, 7.65; S, 8.70.

### 2-Benzylidene-4-(pyrrolidin-1-ylmethyl)-1-thia-4-azaspiro[4.5]decan-3-one (6c))

Yellow crystals; mp 126–127°C; IR: 3020 (CH arom.), 2880 (CH aliph.), 1705 (C = O), 695 (C-S); ^1^H NMR (CDCl_3_): 1.43-1.52 (m, 6H, 3CH_2_), 1.62 (t, 4H, 2CH_2,_*J* = 7.1), 2.17-2.40 (m, 4H, 2CH_2_), 2.53 (t, 4H, 2CH_2,_*J* = 7.1), 4.01 (s, 2H, CH_2_), 7.30-7.58 (m, 5H, Ph-H), 7.60 (s, 1H, CH) ppm; EI-MS, *m*/*z* (%): 341.82 (15) [*M*^*+*^], 258.09 (25), 215.85 (100.0). Calc. for C_20_H_26_N_2_OS: C, 70.14; H, 7.65; N, 8.18; S, 9.36. Found: C, 69.80; H, 7.43; N, 7.76; S, 9.08.

### 2-(4-Chlorobenzylidene)-4-(piperidin-1-ylmethyl)-1-thia-4-azaspiro[4.5]decan-3-one (6d))

Yellow crystals; mp 120–122°C; IR: 3050 (CH arom.), 2955 (CH aliph.), 1700 (C = O), 695 (C-S); ^1^H NMR (CDCl_3_): 1.44–1.61 (m, 12H, 6CH_2_), 2.10-2.46 (m, 8H, 4CH_2_), 4.25 (s, 2H, CH_2_), 7.40 (d, 2H, 2CH, *J* = 7.6), 7.60 (s, 1H, CH), 7.72 (d, 2H, 2CH, *J* = 7.6) ppm; EI-MS, *m*/*z* (%): 390.65 (32) [*M*^*+*^], 292.12 (16), 215.05 (100.0). Calc. for C_21_H_27_ClN_2_OS: C, 64.51; H, 6.96; Cl, 9.07; N, 7.17; S, 8.20. Found: C, 64.22; H, 6.66; Cl, 8.81; N, 7.00; S, 7.92.

### 2-(4-Chlorobenzylidene)-4-(morpholinomethyl)-1-thia-4-azaspiro[4.5]decan-3-one (6e))

Pale yellow crystals; mp 141–143°C; IR: 3050 (CH arom.), 2880 (CH aliph.), 1700 (C = O), 695 (C-S); ^1^H NMR (DMSO-d_6_): 1.44–1.56 (m, 6H, 3CH_2_), 2.18-2.45 (m, 4H, 2CH_2_), 2.52 (t, 4H, 2CH_2_, *J* = 7.8), 3.50 (t, 4H, 2CH_2_, *J* = 7.9), 4.23 (s, 2H, CH_2_), 7.38 (d, 2H, 2CH, *J* = 6.5), 7.64 (s, 1H, CH), 7.70 (d, 2H, 2CH, *J* = 6.5) ppm; EI-MS, *m*/*z* (%): 392.71 (56) [*M*^*+*^], 292.00 (20), 216.65 (100.0). Calc. for C_20_H_25_ClN_2_O_2_S: C, 61.13; H, 6.41; Cl, 9.02; N, 7.13; S, 8.16. Found: C, 60.86; H, 6.20; Cl, 8.80; N, 6.90; S, 7.88.

### 2-(4-Chlorobenzylidene)-4-(pyrrolidin-1-ylmethyl)-1-thia-4-azaspiro[4.5]decan-3-one (6f))

Pale yellow crystals; mp 133–135°C; IR: 3035 (CH arom.), 2895 (CH aliph.), 1700 (C = O), 690 (C-S); ^1^H NMR (CDCl_3_): 1.40-1.51 (m, 6H, 3CH_2_), 1.64 (t, 4H, 2CH_2_, *J* = 7.5), 2.25-2.38 (m, 4H, 2CH_2_), 2.62 (t, 4H, 2CH_2_, *J* = 7.5), 4.12 (s, 2H, CH_2_), 7.35 (d, 2H, 2CH, *J* = 6.3), 7.62 (d, 2H, 2CH, *J* = 6.3), 7.64 (s, 1H, CH) ppm; EI-MS, *m*/*z* (%): 375.90 (18) [*M*^*+*^], 291.89 (30), 215.70 (100.0). Calc. for C_20_H_25_ClN_2_OS: C, 63.73; H, 6.68; Cl, 9.41; N, 7.43; S, 8.51. Found: C, 63.45; H, 6.70; Cl, 9.19; N, 7.20; S, 8.27.

### 2-(4-Bromobenzylidene)-4-(piperidin-1-ylmethyl)-1-thia-4-azaspiro[4.5]decan-3-one (6g))

Pale yellow crystals; mp 133–135°C; IR: 3050 (CH arom.), 2940 (CH aliph.), 1705 (C = O), 690 (C-S); ^1^H NMR (CDCl_3_): 1.38–1.50 (m, 12H, 6CH_2_), 2.05-2.47 (m, 8H, 4CH_2_), 4.05 (s, 2H, CH_2_), 7.31 (d, 2H, 2CH, *J* = 7.1), 7.58 (s, 1H, CH), 7.62 (d, 2H, 2CH, *J* = 7.1) ppm; EI-MS, *m*/*z* (%): 436.15 (19) [M + 2], 434.09 (21) [*M*^*+*^], 335.60 (11), 216.95 (100.0). Calc. for C_21_H_27_BrN_2_OS: C, 57.93; H, 6.25; Br, 18.35; N, 6.43; S, 7.36. Found: C, 57.68; H, 6.00; Br, 18.05; N, 6.22; S, 7.10.

### 2-(4-Bromobenzylidene)-4-(morpholinomethyl)-1-thia-4-azaspiro[4.5]decan-3-one (6h))

Pale yellow crystals; mp 152–153°C; IR: 3030 (CH arom.), 2850 (CH aliph.), 1705 (C = O), 695 (C-S); ^1^H NMR (DMSO-d_6_): 1.40–1.53 (m, 6H, 3CH_2_), 2.10-2.40 (m, 4H, 2CH_2_), 2.52 (t, 4H, 2CH_2_, *J* = 6.9), 3.42 (t, 4H, 2CH_2_, *J* = 6.9), 4.09 (s, 2H, CH_2_), 7.35 (d, 2H, 2CH, *J* = 6.5), 7.65 (s, 1H, CH), 7.58 (d, 2H, 2CH, *J* = 6.5) ppm; EI-MS, *m*/*z* (%): 438.01 (9) [*M*^*+*^ + 2], 436.2 (10) [*M*+], 336.20 (43), 216.75 (100.0). Calc. for C_20_H_25_BrN_2_O_2_S: C, 54.92; H, 5.76; Br, 18.27; N, 6.40; S, 7.33 Found: C, 54.75; H, 5.80; Br, 18.05; N, 6.17; S, 7.09.

### 2-(4-Bromobenzylidene)-4-(pyrrolidin-1-ylmethyl)-1-thia-4-azaspiro[4.5]decan-3-one (6i))

Pale yellow crystals; mp 142–143°C; IR: 3040 (CH arom.), 2890 (CH aliph.), 1700 (C = O), 695 (C-S); ^1^H NMR (CDCl_3_): 1.41-1.50 (m, 6H, 3CH_2_), 1.60 (t, 4H, 2CH_2_, *J* = 7.1), 2.20-2.35 (m, 4H, 2CH_2_), 2.59 (t, 4H, 2CH_2_, *J* = 7.1), 4.10 (s, 2H, CH_2_), 7.33 (d, 2H, 2CH, *J* = 6.5), 7.47 (s, 1H, CH), 7.55 (d, 2H, 2CH, *J* = 6.5) ppm; EI-MS, *m*/*z* (%): 420.96 (26) [*M*^*+*^], 335.89 (45), 216.71 (100.0). Calc. for C_20_H_25_BrN_2_OS: C, 57.00; H, 5.98; Br, 18.96; N, 6.65; S, 7.61. Found: C, 56.15; H, 5.69; Br, 18.80; N, 6.50; S, 7.57.

### 2-(4-Nitrobenzylidene)-4-(piperidin-1-ylmethyl)-1-thia-4-azaspiro[4.5]decan-3-one (6j))

Yellow crystals; mp 142–144°C; IR: 3050 (CH arom.), 2900 (CH aliph.), 1700 (C = O), 690 (C-S); ^1^H NMR (CDCl_3_): 1.45–1.61 (m, 12H, 6CH_2_), 2.17-2.49 (m, 8H, 4CH_2_), 4.26 (s, 2H, CH_2_), 7.92 (s, 1H, CH), 8.14 (d, 2H, 2CH, *J* = 6.8), 8.35 (d, 2H, 2CH, *J* = 6.8) ppm; EI-MS, *m*/*z* (%): 402.16 (15) [*M*^+^ + 1], 303.62 (22), 215.85 (100.0). Calc. for C_21_H_27_N_3_O_3_S: C, 62.82; H, 6.78; N, 10.47; S, 7.99. Found: C, 62.60; H, 6.57; N, 10.49; S, 7.76.

### 4-(Morpholinomethyl)-2-(4-nitrobenzylidene)-1-thia-4-azaspiro[4.5]decan-3-one (6k))

Yellow crystals; mp 158–160°C; IR: 3020 (CH arom.), 2900 (CH aliph.), 1700 (C = O), 695 (C-S); ^1^H NMR (DMSO-d_6_): 1.45–1.58 (m, 6H, 3CH_2_), 2.20-2.52 (m, 4H, 2CH_2_), 2.55 (t, 4H, 2CH_2_, *J* = 7.5), 3.54 (t, 4H, 2CH_2_, *J* = 7.5), 4.20 (s, 2H, CH_2_), 7.71 (s, 1H, CH), 8.08 (d, 2H, 2CH, *J* = 6.8), 8.29 (d, 2H, 2CH, *J* = 6.8) ppm; EI-MS, *m*/*z* (%): 403.12 (41) [*M*^*+*^], 303.25 (35), 215.36 (100.0). Calc. for C_20_H_25_N_3_O_4_S: C, 59.53; H, 6.25; N, 10.41; S, 7.95. Found: C, 59.58; H, 6.01; N, 10.26; S, 7.71.

### 2-(4-Nitrobenzylidene)-4-(pyrrolidin-1-ylmethyl)-1-thia-4-azaspiro[4.5]decan-3-one (6l))

Yellow crystals; mp 149–151°C; IR: 3025 (CH arom.), 2990 (CH aliph.), 1705 (C = O), 690 (C-S); ^1^H NMR (CDCl_3_): 1.43-1.52 (m, 6H, 3CH_2_), 1.61 (t, 4H, 2CH_2_, *J* = 6.9), 2.25-2.37 (m, 4H, 2CH_2_), 2.58 (t, 4H, 2CH_2_, *J* = 6.9), 4.20 (s, 2H, CH_2_), 7.41 (s, 1H, CH), 7.93 (d, 2H, 2CH, *J* = 6.8), 8.21 (d, 2H, 2CH, *J* = 6.8) ppm; EI-MS, *m*/*z* (%): 387.20 (19) [*M*^*+*^], 303.10 (44), 215.87 (100.0). Calc. for C_20_H_25_N_3_O_3_S: C, 61.99; H, 6.50; N, 10.84; S, 8.27. Found: C, 62.05; H, 6.24; N, 10.70; S, 8.00.

### 2-(4-Methoxybenzylidene)-4-(piperidin-1-ylmethyl)-1-thia-4-azaspiro[4.5]decan-3-one (6m))

Pale yellow crystals; mp 121–123°C; IR: 3200 (NH), 3090 (CH arom.), 1700 (C = O), 690 (C-S); ^1^H NMR (CDCl_3_): 1.42–1.53 (m, 12H, 6CH_2_), 2.15-2.41 (m, 8H, 4CH_2_), 3.50 (s, 3H, CH_3_), 4.10 (s, 2H, CH_2_), 6.92 (d, 2H, 2CH, *J* = 6.8), 7.45 (d, 2H, 2CH, *J* = 6.8), 7.56 (s, 1H, CH) ppm; EI-MS, *m*/*z* (%): 386.20 (15) [M^+^], 288 (45), 216.05 (100.0). Calc. for C_22_H_30_N_2_O_2_S: C, 68.36; H, 7.82; N, 7.25; S, 8.30. Found: C, 68.39; H, 7.70; N, 7.00; S, 8.02.

### 2-(4-Methoxybenzylidene)-4-(morpholinomethyl)-1-thia-4-azaspiro[4.5]decan-3-one (6n))

Pale yellow crystals; mp 133–135°C; IR: 3050 (CH arom.), 2950 (CH aliph.), 1700 (C = O), 695 (C-S); ^1^H NMR (CDCl_3_): 1.41–1.52 (m, 6H, 3CH_2_), 2.15-2.46 (m, 4H, 2CH_2_), 2.51 (t, 4H, 2CH_2_, *J* = 7.1), 3.50 (t, 4H, 2CH_2_, *J* = 7.1), 4.08 (s, 2H, CH_2_), 7.01 (d, 2H, 2CH, *J* = 6.9), 7.60 (d, 2H, 2CH, *J* = 6.9), 7.69 (s, 1H, CH) ppm; EI-MS, *m*/*z* (%): 388.19 (20) [*M*^*+*^], 288.75 (51), 215.06 (100.0). Calc. for C_21_H_28_N_2_O_3_S: C, 64.92; H, 7.26; N, 7.21; S, 8.25. Found: C, 64.70; H, 7.00; N, 7.30; S, 8.01.

### 2-(4-Methoxybenzylidene)-4-(pyrrolidin-1-ylmethyl)-1-thia-4-azaspiro[4.5]decan-3-one (6o))

Yellow crystals; mp 140–142°C; IR: 3050 (CH arom.), 2900 (CH aliph.), 1700 (C = O), 695 (C-S); ^1^H NMR (CDCl_3_): 1.40-1.49 (m, 6H, 3CH_2_), 1.60 (t, 4H, 2CH_2_), 2.20-2.32 (m, 4H, 2CH_2_), 2.55 (t, 4H, 2CH_2_, *J* = 7.3), 4.15 (s, 2H, CH_2_, *J* = 7.3), 7.01 (d, 2H, 2CH, *J* = 6.8), 7.26 (d, 2H, 2CH, *J* = 6.8), 7.29 (s, 1H, CH) ppm; EI-MS, *m*/*z* (%): 372.19 (32) [*M*^*+*^], 289.02 (35), 215.97 (100.0). Calc. for C_21_H_28_N_2_O_2_S: C, 67.71; H, 7.58; N, 7.52; S, 8.61. Found: C, 67.58; H, 7.60; N, 7.40; S, 8.45.

### 4-(Piperidin-1-ylmethyl)-2-(pyridin-4-ylmethylene)-1-thia-4-azaspiro[4.5]decan-3-one (6p))

Pale yellow crystals; mp 111–113°C; IR: 3050 (CH arom.), 1695 (C = O), 690 (C-S); ^1^H NMR (CDCl_3_): 1.40–1.56 (m, 12H, 6CH_2_), 2.01-2.55 (m, 8H, 4CH_2_), 4.00 (s, 2H, CH_2_), 7.05 (d, 2H, 2CH, *J* = 6.9), 7.30 (s, 1H, CH), 7.83 (d, 2H, 2CH, *J* = 6.9), ppm; EI-MS, *m*/*z* (%): 357.15 (16) [*M*+], 259.30 (40), 217.15 (100.0). Calc. for C_20_H_27_N_3_OS: C, 67.19; H, 7.61; N, 11.75; S, 8.97. Found: C, 67.25; H, 7.50; N, 11.80; S, 8.66.

### 4-(Morpholinomethyl)-2-(pyridin-4-ylmethylene)-1-thia-4-azaspiro[4.5]decan-3-one (6q))

Pale yellow crystals; mp 125–127°C; IR: 3050 (CH arom.), 1700 (C = O), 695 (C-S); ^1^H NMR (CDCl_3_): 1.39–1.50 (m, 6H, 3CH_2_), 2.15-2.45 (m, 4H, 2CH_2_), 2.52 (t, 4H, 2CH_2_, *J* = 7.1), 3.47 (t, 4H, 2CH_2_, *J* = 7.1), 4.10 (s, 2H, CH_2_), 6.91 (d, 2H, 2CH, *J* = 6.9), 7.15 (s, 1H, CH), 7.80 (d, 2H, 2CH, *J* = 6.9) ppm; EI-MS, *m*/*z* (%): 359.60 (21) [*M*+], 259.95 (61), 216.95 (100.0). Calc. for C_19_H_25_N_3_O_2_S: C, 63.48; H, 7.01; N, 11.69; S, 8.92. Found: C, 63.20; H, 6.85; N, 11.71; S, 8.70.

### 2-(Pyridin-4-ylmethylene)-4-(pyrrolidin-1-ylmethyl)-1-thia-4-azaspiro[4.5]decan-3-one (6r))

Pale yellow crystals; mp 119–120°C; IR: 3050 (CH arom.), 1695 (C = O), 690 (C-S); ^1^H NMR (CDCl_3_): 1.40-1.49 (m, 6H, 3CH_2_), 1.59 (t, 4H, 2CH_2_, *J* = 7.2), 2.22-2.40 (m, 4H, 2CH_2_), 2.55 (t, 4H, 2CH_2_, *J* = 7.2), 4.08 (s, 2H, CH_2_), 6.98 (d, 2H, 2CH, *J* = 6.4), 7.20 (s, 1H, CH), 7.90 (d, 2H, 2CH, *J* = 6.4) ppm; EI-MS, *m*/*z* (%): 343.05 (20) [*M*+], 259.02 (65), 216.65 (100.0). Calc. for C_19_H_25_N_3_OS: C, 66.44; H, 7.34; N, 12.23; S, 9.34. Found: C, 66.50; H, 7.36; N, 12.00; S, 9.16.

### Antimicrobial screening

#### Antibacterial activity

The newly synthesized compounds were screened for their antibacterial activity against bacterial isolate namely *Staphylococcus aureus* (ATCC 29213) as Gram positive bacteria, *Escherichia Coli* (ATCC 25922), *Pseudomonas aeroginosa* (ATCC 27953) as Gram negative bacteria by cup-plate method [23]. The sterilized nutrient agar medium was distributed 100 ml each in two 250 ml conical flasks and allowed to cool to room temperature. To these media, 18–24 h grown bacterial subcultures were added and shaken thoroughly to ensure uniform distribution of organism throughout the medium. Then, this agar medium was distributed in equal portions, in sterilized Petri dishes, ensuring that each Petri dish contains about 20 ml of the medium. The medium was then allowed for solidification. Then, cups were made with the help of a sterile cork borer (6 mm diameter) punching into the set of agar media.

The solutions of required concentration (50 μg/mL) of test compounds were prepared by dissolving the compounds in DMSO were filled into the cups with 1 mL of respective solution. Then, the Petri dishes were kept for incubation in an inverted position for 24–48 h at 37°C in an incubator. When growth inhibition zones were developed surrounding each cup, their diameter in cm was measured and compared with that of the Ciprofloxacin.

#### Antifungal activity

The newly synthesized compounds were screened for their antifungal activity against three fungal strains; *Aspergillus niger*, *Candida albicans, Fusarium oxysporium* at the concentration levels of 50 μg/mL by cup-plate method, using Nystatin as the standard. To the sterilized potato dextrose agar medium incubated for 72 h, subculture of fungus were added and shaken thoroughly to ensure uniform distribution. Then, this was poured into previously sterilized and labeled Petri dishes and allowed to solidify. Then, with the help of a borer four cups were made in each plate. Two cups were filled with 0.1 ml of two test dilutions and the other two cups with respective concentrations of standard dilutions. Then, the plates were left as it is for 2–3 h for diffusion and then they were kept for incubation at 37°C for 24 h. Then the diameter of the zones of growth inhibition was measured and compared with that of standard.
